# Genome-wide copy number variation (CNV) in patients with autoimmune Addison's disease

**DOI:** 10.1186/1471-2350-12-111

**Published:** 2011-08-18

**Authors:** Ingeborg Brønstad, Anette SB Wolff, Kristian Løvås, Per M Knappskog, Eystein S Husebye

**Affiliations:** 1 Institute of Medicine, University of Bergen, 5021 Bergen, Norway; 2 Department of Medicine, Haukeland University hospital, 5021 Bergen, Norway; 3 Institute of Clinical Medicine, University of Bergen, 5021 Bergen, Norway; 4 Department of Medical Genetics, Haukeland University hospital, 5021 Bergen, Norway

## Abstract

**Background:**

Addison's disease (AD) is caused by an autoimmune destruction of the adrenal cortex. The pathogenesis is multi-factorial, involving genetic components and hitherto unknown environmental factors. The aim of the present study was to investigate if gene dosage in the form of copy number variation (CNV) could add to the repertoire of genetic susceptibility to autoimmune AD.

**Methods:**

A genome-wide study using the Affymetrix GeneChip^® ^Genome-Wide Human SNP Array 6.0 was conducted in 26 patients with AD. CNVs in selected genes were further investigated in a larger material of patients with autoimmune AD (n = 352) and healthy controls (n = 353) by duplex Taqman real-time polymerase chain reaction assays.

**Results:**

We found that low copy number of *UGT2B28 *was significantly more frequent in AD patients compared to controls; conversely high copy number of *ADAM3A *was associated with AD.

**Conclusions:**

We have identified two novel CNV associations to *ADAM3A *and *UGT2B28 *in AD. The mechanism by which this susceptibility is conferred is at present unclear, but may involve steroid inactivation (*UGT2B28*) and T cell maturation (*ADAM3A*). Characterization of these proteins may unravel novel information on the pathogenesis of autoimmunity.

## Background

Primary adrenal insufficiency (Addison's disease (AD)) is most commonly caused by an autoimmune destruction of the adrenal cortex, resulting in failure to produce corticosteroids. AD can appear alone, but is frequently part of autoimmune polyendocrine syndromes (APS). APS type I is caused by mutations in the gene autoimmune regulator (*AIRE*), which has a function in developing immunological tolerance in the thymus (for review see [1]). Isolated AD, or AD in combination with other endocrine components where APS I has been ruled out, is thought to be caused by a combination of genetic and environmental factors, and stochastic events.

Using the candidate gene approach, several genes have been associated with autoimmune AD. The human leukocyte antigen (HLA) haplotypes *DR3-DQ2 *and *DR4-DQ8 *is the strongest predisposing genetic factors [2,3]. Other susceptibility genes are *MHC-class I related chain A *and *B *(*MICA *and *MICB*) [4,5], *cytotoxic T lymphocyte antigen-4 (CTLA-4) *[6], *tyrosine-protein phosphatase non-receptor type 22 *(*PTPN22) *[7], the gene encoding MHC class II transactivator (*CIITA) *[8], *C-type lectin domain family 16, member A (CLEC16A*) [9], *Programmed death ligand 1 (PD-L1) *[10], and the gene coding for NACHT leucine rich repeats protein 1 (*NALP1*) [11]. All these genes are also associated with other autoimmune diseases and can be viewed as autoimmunity genes. So far a disease-specific AD gene has not been identified, with the possible exception of the DRB1*0404 HLA-subtype [2].

The recent identification of common copy number variation (CNV) in the human genome as a source of genetic modification could possibly explain some of the susceptibility to autoimmunity. In support of this notion is the finding that low copy number of *FCGR3B *(Fc fragment of IgG, low affinity IIIb, receptor (CD16b)), has been coupled to the systemic autoimmune diseases systemic lupus erythematosus (SLE), vasculitis, microscopic polyangiitis and Wegener's granulomatosis [12]. Low *FCGR3B *copy number possibly contributes to impaired clearance of immune complexes, which is of pathogenic importance in these diseases [12]. Organ-specific diseases like Graves' disease and AD, on the other hand, fail to show this association [12]. A similar gene, *FCGR2C*, is associated with idiopathic thrombocytopenic purpura [13]. Furthermore, low copy number of the complement associated genes *C4A *and *C4B *have been described in SLE-patients (reviewed in [14]). Finally, aberrant copy number of the gene *chemokine ligand 3-like 1 *(*CCL3L1*), a potent binder of several pro-inflammatory cytokine receptors, has been reported in SLE [15], while high copy number has been reported in rheumatoid arthritis and type 1 diabetes [16].

These studies show that CNVs in immunoregulatory genes could add to the susceptibility to autoimmune diseases, and that identification of such genes can contribute to the understanding of pathogenesis. Only a few reports on genome-wide CNV have been reported in organ-specific autoimmune diseases so far [17]. Here, we have conducted the first genome-wide (GW) CNV study in autoimmune AD.

## Methods

### Subjects

**Figure 1:**
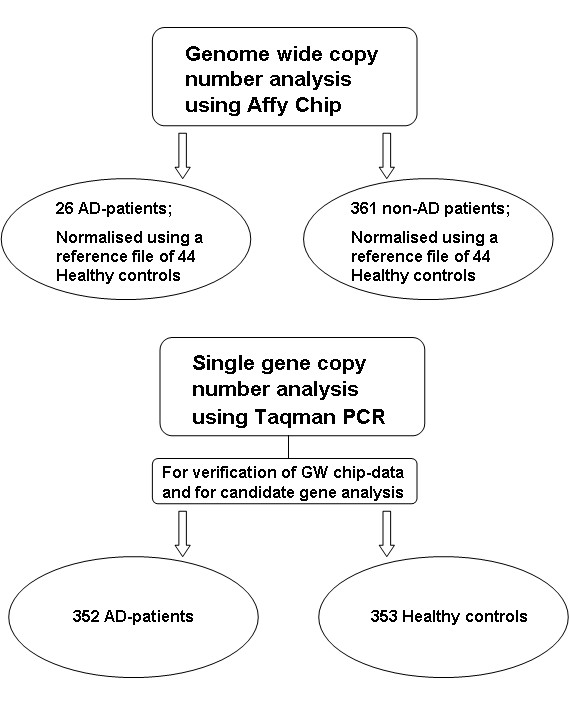
**Flow chart of the methods and subjects used in the present study**.

A total of 352 patients with autoimmune AD (63% females, 37% males; mean age 57 years, range 13 - 93 years; 52% of whom had APS type II) recruited from the National Norwegian Registry of patients with AD [2], and 353 blood donors (35% females, 65% males, mean age 46 years, range 19 - 71 years) were available for CNV analyses. For comparison we had access to an in-house database with CNV-results from 361 non-Addison's patients analysed with the same GW array. These samples comprise individuals which were analysed for CNVs in the daily routine analyses at the Medical Genetics Department, Haukeland University Hospital, for a diversity of dysfunctions. An overview of the subjects used in the present study is given in Figure 1.

### Ethical considerations

All included patients and blood donors signed a written consent form. The study was approved by the Regional Committee for Medical Ethics of Western Norway, and performed according to the Helsinki Declaration.

### GW CNV gene chip analyses

Twenty eight unrelated patients with isolated autoimmune AD (39% females, 61% males, mean age 63 years, ranging from 26 to 96 years) were randomly selected for GW CNV analyses. The genome-wide copy number screening was performed using the Affymetrix GeneChip^® ^Genome-Wide Human SNP Array 6.0, Part# 901182, (Affy 6.0) following the procedure described in Affymetrix^® ^Cytogenetics Copy Number User Guide. The arrays were scanned by the Gene Chip Scanner 3000 7G, controlled by the AGCC software. The array data were analyzed by the Affymetrix Genotyping Console™ version 3.0.1. The Contrast quality control (QC) was set to higher than 0.4 and the Median Absolute Pair wise Difference (MAPD) threshold was set to 0.30 or less for each sample. CNV analysis was performed by comparing the files from the AD patients with a reference file of 44 healthy controls (anonymous material used in the daily routine analyses at the Medical Genetics Department, Haukeland University Hospital). CNV were further evaluated in the Affymetrix^® ^Genotyping Console Browser version 1.0.11 and the Affymetrix Chromosome Analysis Suite version 1.0.1. The CNVs were flagged using a cut off filter of minimum 20 markers per 100 Kbp segment. Flagged CNVs were checked against the Database of Genomic Variants (DGV) (http://projects.tcag.ca/variation/)©[18]. Findings that varied from the reference file were compared with the in-house database from 361 non-AD individuals. In addition, a search for CNVs in candidate genes of Addison's disease was performed on the chip data without using cut off filters (see Additional file 1).

### Additional CNV assays

Individual CNV assays were performed by duplex Taqman real-time PCR assays in order to confirm findings on new candidates from the GW analysis in a larger patient and blood donor material. In addition, an individual CN assay was conducted for *FCGR3B *since it is not included on the Affy 6.0 chip. *FCGR3B, KIAA1267 *and *ADAM3A *copy number assays were supplied from Applied Biosystems (Assay ID: Hs04211858_cn, Hs03983160_cn, and Hs03269461_cn, respectively). Copy number assay for UGT2B28 was designed by Applied Biosystems based on the gene's sequence (ugt2b28_CCGJPAM). *RNaseP *(TaqMan copy number reference assay, part # 4403328) and *Desmoplakin *(*DSP) *(adapted from Parajes et al, 2007) [19] were used as reference genes. All primers and probes were supplied from Applied Biosystems, and duplex real-time PCR assays were performed according to the TaqMan^® ^copy number assay protocol (Applied Biosystems). All data was further analyzed using the 7300 System SDS software version 2.3. The genes were quantified by calibration curves for each gene. The reference genes are known to occur in two copies in the genome. Hence, the copy number was determined by the relative relationship between the quantity of the candidate gene and the reference gene.

### Statistics

Fisher's Exact test was applied for comparing the frequencies of CNs of the different genes in AD patients and controls, using the statistical work package PASW Statistics 18. The data was considered to be significant when P < 0.05.

## Results

### Quality control of the Affy 6.0 gene chips

Twenty-six of twenty-eight arrays in the patient group and all controls (N = 44) were in accordance with Affymetrix' recommendations, with MAPD values ranging from 0.194-0.299 and QC call rate ranging from 88.2-99.0%. Two patient arrays failed these quality criteria and were excluded from further analyses.

### Frequent CNVs associated with AD

The gene regions with most frequent CNVs in AD patients are given in Table 1 all of which are reported as normal CN polymorphisms in the DGV [18]. Except for the *DEFB *cluster region, a number of genes within this region were found to have CNV that differed significantly from 361 in-house non-AD controls (Table 1). Low CN of *KIAA1267 *(coding for an uncharacterised protein) and *UGT2B28 *(encoding uridine diphosphate glucuronosyltransferase (UGT) 2 family, polypeptide B28), and high CN of *ADAM3A *(encoding a disintegrin and metalloproteinase domain 3A), occurred more frequently (> 30%) in AD patients. These were selected for replication studies in the large Addison and control cohorts.

**Table 1:** Gene regions (≥ 100 Kbp) of the most frequent copy number (CN) variation in patients with Addison's disease detected by gene chip.

Patient ID	CN	Chromosome	Genes	CN Frequency (%)^1^		P-value^2^
					
				AD n = 26	Controls n = 361	
14, 17, 19, 20	1 3 ≥ 4	1q21.1	*FCGR1C*	15.3 0 0	2.2 0.8 0.3	P = 0.012

22 10, 19, 20, 26 2, 3	0 1 3	4q13.2	*UGT2B17*	3.8 15.3 7.7	1.9 1.4 5.0	P = 0.0018

8, 9, 13, 14, 15, 19, 22, 26	0 1	4q13.2	*UGT2B28*	0 30.7	0.3 10	P = 0.01

11, 16, 21, 26 19, 22 2, 5, 9, 10, 12, 13, 15, 17, 20	0 3 ≥ 4	8p11.23-11.22	*ADAM3A*	15.4 7.7 34.6	3.6 0.3 9.7	P = 3.3 × 10^-7^

9, 14, 21, 23	1 3	8p23.1	*DEFB cluster*	15.3 0	5.8 2.2	P = 0.165

5, 8, 10, 13, 16, 19, 20, 22, 23, 24 12, 15, 26	1 3 ≥ 4	17q21.31	*KIAA1267*	38.4 11.5 0	8.5 3.3 0.6	P = 3.3 × 10^-6^

The exact size and localisation of the CNVs varied slightly between patients, but in every case encompassed the listed genes.

^1^CN frequency (%) is the percentage of the individuals with the given copy number. ^2^CN frequencies in patients with Addison's disease (AD) were compared to controls and P-values for significance were calculated by Fisher's Exact test.

**Figure 2:**
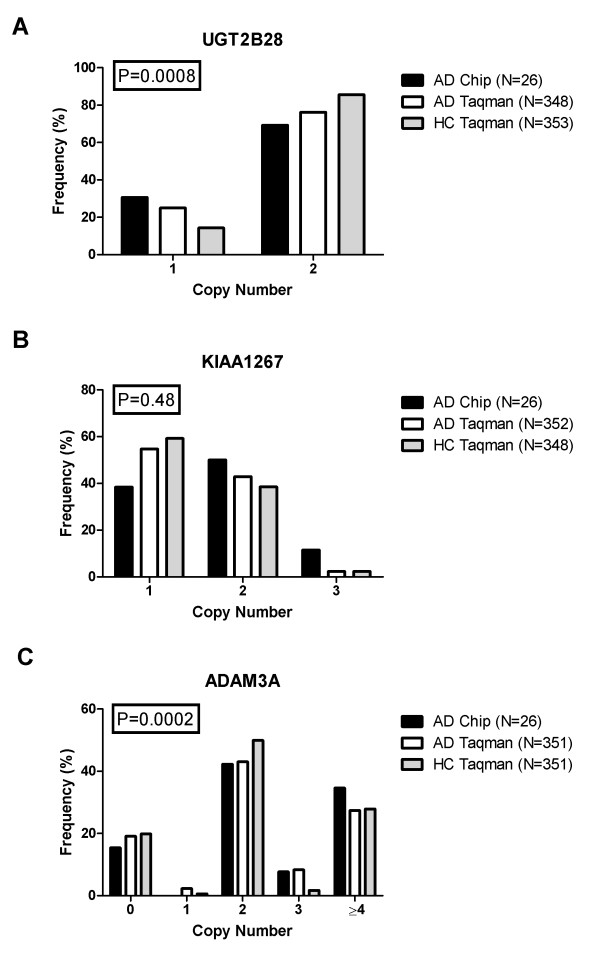
**Copy number variations in genes associated with AD by the Affy 6.0 chip**. Copy number frequencies in Addison patients and controls for the three genes associated with AD by the Affy 6.0 chip (AD chip) and validated by duplex RT-qPCR. *UGT2B28 *(a), *KIAA1267 *(b), and *ADAM3A *(c). P-values for differences of copy number variation between Addison patients (AD Taqman) and healthy controls (HC Taqman) were calculated by Fishers's Exact test.

The Affy 6.0 gene chip experiment revealed that a heterozygous deletion (CN = 1) in the *UGT2B28 *gene occurred more often in the AD patients (30%) than in the in-house reference material (10%). This association was confirmed (25% vs 14% P = 0.0008) in the large AD patient and blood donor materials using the gene specific CN assay (Figure 2a). On the other hand, the heterozygous deletion (CN = 1) in the *KIAA1267 *gene found to be more frequently in AD patients (38% vs 9% respectively, Table 1), was no longer significant (55% vs 59%, P = 0.48) (Figure 2b) in the replication study.

High copy numbers (CN > 2) of the *ADAM3A *gene were detected more frequently in AD patients compared to the in-house reference database (42% vs 10% respectively) by the Affy 6.0 gene chip analysis (Table 1). A significant association (P = 0.0002) of CNVs, with respect to CN = 3, was still evident in the replication experiment on the larger cohort with duplex Taqman real-time PCR assays (Figure 2c).

The differences in CNVs for *UGT2B28 *and *ADAM3A *were still evident when patients with isolated AD and APS II were analysed separately. With respect to sex, the association to *UGT2B28 *was only significant for males (P = 0.002), while the association to *ADAM3A *was significant for both females (P = 0.004) and males (P = 0.004).

### Rare CNV in AD patients detected by Affy 6.0

We also performed a GW scan for rare CNVs (Table 2). These CNVs are not listed in the DGV [18], nor detected in our in-house database. Eight novel CNV gene regions were found in the AD patients, with the longest segment consisting of a 700 Kbp heterozygote deletion which was found in patient #23 on chromosome 7p14.3. This segment covers only one gene, *BMP binding endothelial regulator (BMPER)*, an inhibitor of bone morphogenetic protein (BMP) function. Patient #3 had two large CNVs regions, one heterozygote deletion of 103 Kbp in 5q23.1 covering the last third part of the 17*-beta-hydroxysteroid dehydrogenase IV gene (HSD17B4)*, and a single copy gain of 115 Kbp in 14q13.2, covering the *NFKBIA *gene *(nuclear factor of kappa light polypeptide gene enhancer in B-cells inhibitor, alpha)*. Patient #20 had two regions of single copy gain, one in 3q21.3 (102 Kbp), and one in 8p21.1 (103 Kbp) covering the gene *SCARA5 *(*Scavenger receptor class A, member 5*).

**Table 2:** Gene regions of rare copy number (CN) variation in patients with Addison's disease detected by gene chip.

Patient ID	CN	Chromosome	Genes	Markers start/end (Marker count)	Size Kbp
4	1	2q22.1	*LRP1B*	SNP A-2084015/SNP A-2042103 (88)	135
14	1	3p24.3	*TBC1D5*	SNP A-2076685/CN 981261 (46)	100
20	3	3q21.3	*H1FOO, RHO, PLXND1*	CN 1010717/CN 1010739 (52)	102
3	1	5q23.1	*HSD17B4*	CN 1103972/SNP A-8663851 (71)	103
23	1	7p14.3	*BMPER*	CN 1244766/SNP A-8598772 (491)	700
20	3	8p21.1	*SCARA5*	CN 1292435/SNP A-8280696 (82)	103
1	1	12q21.31	*RASSF9*	SNP A-4273275/CN 608032 (202)	312
3	3	14q13.2	*NFKBIA*	SNP A-2263171/CN 662105 (69)	115

### CNV in candidate genes of AD

A number of candidate autoimmune genes of which some have been reported to have CNV associated with autoimmunity (e.g. *FCGR3B*), were tested for association to AD. Of these, only CNVs in the candidate genes *CCL3L1 *and *HLA-DRB5 *were detected by the gene chip (see Additional file 1). No significant differences in CNV were found in the selected candidates, including *FCGR3B *(see Additional file 2).

## Discussion

The present study is the first effort to search for common CNVs as a source for genetic susceptibility in AD. We found two novel CNVs associated with AD, *UGT2B28 *and *ADAM3A*. Both have previously been reported as regions with copy number polymorphisms [18].

The enzyme UGT2B28 is expressed in liver and mammary glands, and it might play a role in steroid inactivation [20]. In the present study we found an increased frequency of CN = 1 of the gene *UGT2B28 *in our AD cohort. It has previously been reported that this heterozygote deletion occurs in 23% of healthy Caucasians, and deletions in this gene region, which also includes the *UGT2B17 *gene, are very common [21]. The connection to autoimmunity, if any, is at present not clear.

We also found an association with CNV and *ADAM3A *in AD patients. Three different transcript variants of *ADAM3A *(Entrez Gene; http://www.ncbi.nlm.nih.gov/gene; Gene ID 1587) have been reported, and the gene is embedded in a cluster of other genes in the *ADAM *family [18]. Several proteins of the ADAM family are believed to be involved in the inflammation process, and recent studies have demonstrated their role in T cell maturation and development [22,23]. The function of *ADAM3A *has mainly been associated with sperm-egg binding and fusion [24], but it has also been linked to pro-inflammatory activity and survival of macrophages [25]. Thus a role in autoimmunity and immunological tolerance can be envisioned.

Recent evidence shows that common genetic variants do not explain all genetic susceptibility to autoimmune diseases. Rare variants in a number of genes, e.g. *PTPN22*, have been shown to be associated with autoimmune diseases such as AD [7] and diabetes [26-28]. One could hypothesize that rare CNV could play a role for AD susceptibility. Thus, as an alternative approach we looked for rare or private CNVs. We found that one patient had a single copy gain of the *NFKBIA *gene, previously shown to be related to Crohn's diseases [29] and multiple sclerosis [30]. This gene produces an inhibitor of *NFKB1*, which encodes the NFκB subunit 1. Gene polymorphisms in NFκB have been associated with type 1 diabetes [31]. The same patient also had a heterozygous deletion in the *HSD17B4 *gene. Mutations and deletions in this gene have been reported to cause D-bifunctional protein deficiency (http://www.ncbi.nlm.nih.gov/omim ID 601860). High copy number of the gene *SCARA5 *was also detected in one patient. This gene might be of interest because of its expression in murine thymus and adrenals, and its function in innate immune activities of epithelial cells [32].

When searching for CNVs in candidate gene regions, we only detected aberrant CN in the *CCL3L1 *and *HLA-DRB5 *genes in some of the AD patients, but not significantly different from controls. CNVs of these two genes have been also reported previously as normal variants [18]. Moreover, we did not find any association between CNVs in the *FCGR3B *gene and AD, which is in agreement to the results of Fanciulli *et al *(2007) [12].

## Conclusions

We have identified two novel CNV associations to *ADAM3A *and *UGT2B28 *in AD. The mechanism by which this susceptibility is conferred is at present unclear, but may involve steroid inactivation (*UGT2B28*) and T cell maturation (*ADAM3A*). Characterization of these proteins may unravel novel information on the pathogenesis of autoimmunity.

## Competing interests

The authors declare that they have no competing interests.

## Authors' contributions

IB carried out the genome-wide CN study and the real-time PCR copy number assays, and performed the statistical analysis. ABSW and ESH were responsible for supplying the DNA material from AD patients and healthy controls. KL performed the statistical design and analysis. PMK was responsible for coordinating the gen chip analysis and supplying data from gene chip controls. All the authors have contributed to design the experiment, participated in writing the manuscript, critically read and approved the final manuscript.

## Pre-publication history

The pre-publication history for this paper can be accessed here:

http://www.biomedcentral.com/1471-2350/12/111/prepub

## Supplementary Material

Additional file 1 **Candidate genes of Addison's disease**. List of selected candidate genes of Addison's disease, and CNVs in candidate genes detected by the Affymetrix 6.0 gene chip. Click here for file

Additional file 2 **Copy number variations in *FCGR3B *determined by duplex RT-qPCR**. Copy number frequencies of the *FCGR3B *gene in AD patients and healthy controls. P-values for differences of copy number variation between Addison patients (AD Taqman) and healthy controls (HC Taqman) were calculated by Fishers's exact test. Click here for file
