# Supplementary material for: Genome-wide copy number variation (CNV) in patients with autoimmune Addison's disease
Source: BMC Med Genet. 2011 Aug 18;12:111. doi: 10.1186/1471-2350-12-111 (PMC3166911; doi:10.1186/1471-2350-12-111)
Supplement: Additional file 2 — Copy number variations in FCGR3B determined by duplex RT-qPCR. Copy number frequencies of the FCGR3B gene in AD patients and healthy controls. P-values for differences of copy number variation between Addison patients (AD Taqman) and healthy controls (HC Taqman) were calculated by Fishers's exact test. [file 1471-2350-12-111-S2.PDF]

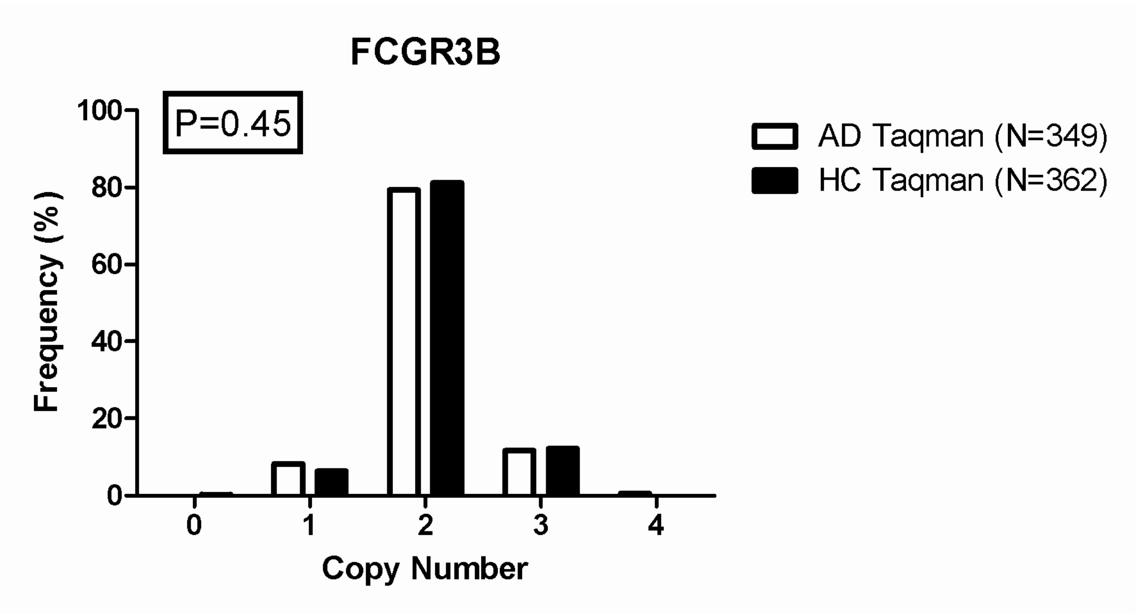

Copy number variations in *FCGR3B* determined by duplex RT-qPCR

Copy number frequencies of the *FCGR3B* gene in AD patients and healthy controls. P-values for differences of copy number variation between Addison patients (AD Taqman) and healthy controls (HC Taqman) were calculated by Fishers’s exact test.
